# Uterus-Preserving Surgery for Placenta Previa and Placenta Accreta Spectrum: Retrospective Analysis of the Evolution of a Standardized Step-Up Approach in a 13-Year Single-Surgeon Cohort of 324 Cases

**DOI:** 10.3390/medicina62081552

**Published:** 2026-08-12

**Authors:** Huseyin Durukan, Kasim Akay

**Affiliations:** 1 Department of Obstetrics and Gynecology, Faculty of Medicine, Mersin University, 33110 Mersin, Turkey; huseyindurukan@gmail.com; 2 Department of Obstetrics and Gynecology, Osmaniye Düziçi State Hospital, 80600 Osmaniye, Turkey

**Keywords:** placenta previa, placenta accreta spectrum, fertility-preserving surgery, peripartum hysterectomy, internal iliac artery ligation, maternal morbidity

## Abstract

*Background and Objectives:* This retrospective cohort study aimed to analyze the surgical evolution, clinical outcomes, and adoption of uterus-preserving techniques in placenta previa (PP) and placenta accreta spectrum (PAS) cases managed by a single senior surgeon over an uninterrupted 13-year period. *Materials and Methods:* A total of 324 patients who underwent cesarean section by a single experienced surgeon with a diagnosis of placenta previa between 1 January 2013 and 1 January 2026 were included in the study. The demographic and obstetric characteristics of the patients, the surgical methods applied, perioperative blood product requirements, complications, and neonatal outcomes were evaluated retrospectively. Uterus-preserving surgical methods included a temporary uterine tourniquet, intrauterine hemostatic sutures, bilateral internal iliac artery ligation (IIAL), Bakri balloon tamponade, B-Lynch suture, uterine lower segment resection, and uterine packing, applied in a stepwise manner. The perioperative hemoglobin change (ΔHb) was calculated as an objective indicator of blood loss. *Results:* Placenta previa constituted a substantial proportion of all cesarean sections performed by the same surgeon during the study period. Total previa was the predominant type, and almost half of the operations were carried out under emergency conditions. Peripartum hysterectomy was required in a minority of patients, and placenta accreta spectrum was histopathologically confirmed in most of these hysterectomy specimens. The proportion of cases managed with uterus-preserving techniques increased progressively across the series, rising from none in the first year to the great majority of cases in the later years, while the peripartum hysterectomy rate declined correspondingly. On multivariable analysis, a greater number of previous cesarean sections independently increased, and operation during the later years of the series independently reduced the likelihood of peripartum hysterectomy. Perioperative transfusion of at least one blood product was required in more than half of the patients, whereas intensive care admission and perioperative complications were infrequent; bladder perforation was the most common complication. Compared with women whose uterus was preserved, those undergoing hysterectomy were older and had markedly longer operative times, greater blood product consumption, longer hospital stays, and a substantially higher complication rate. No maternal death occurred throughout the 13-year period. *Conclusions:* In this 13-year single-surgeon experience, the gradual adoption of uterus-preserving techniques was sustainably associated with low maternal morbidity and zero maternal mortality. The markedly higher complication burden observed in the hysterectomy group compared with the uterus-preserving group demonstrates that a standardized step-up surgical approach offers an applicable and effective management strategy even in resource-limited centers.

## 1. Introduction

Placenta previa is defined as implantation of the placenta in the lower uterine segment, such that placental tissue covers or lies in close proximity to the internal cervical os beyond 20 weeks of gestation. Its estimated incidence at term is approximately 1 in 200 pregnancies, although reported rates vary considerably according to the diagnostic criteria applied and the prevalence of the underlying risk factors [1,2]. Advanced maternal age, multiparity, previous uterine curettage, assisted reproductive technology and, most importantly, a history of previous cesarean delivery are the principal predisposing factors [3,4]. Historically, placenta previa was subclassified as total, partial, marginal, or low-lying according to the relationship between the placental edge and the internal os; following the 2014 joint Fetal Imaging Workshop, this four-tier system was replaced by a binary terminology in which any placenta overlying the internal os is designated placenta previa, whereas a placenta whose edge lies within 2 cm of but does not cover the os is designated low-lying [5]. Clinically, placenta previa is the leading cause of painless antepartum hemorrhage in the second half of pregnancy, mandates cesarean delivery, and carries a substantial risk of major peripartum hemorrhage. Beyond this intrinsic morbidity, placenta previa also constitutes the single most important obstetric setting in which placenta accreta spectrum arises, since implantation over a previous cesarean scar in a poorly decidualized lower uterine segment predisposes to abnormally invasive placentation [2,3].

Placenta accreta spectrum (PAS) is a pathological condition characterized by abnormal adherence or invasion of chorionic villi into the myometrium when the decidua basalis is damaged, and it is one of the leading causes of maternal morbidity and mortality [6]. Historically rare, the incidence of this condition has shown a dramatic increase in recent years, in parallel with rising cesarean rates worldwide [7]. Current epidemiological data reveal that a history of previous cesarean section and placenta previa are the strongest determinants among PAS risk factors; in particular, the risk has been reported to increase exponentially as the number of previous cesarean sections rises [3,8].

Histopathologically, PAS is associated with excessive invasion of extravillous trophoblasts into the uterine wall and a decidualization defect at the implantation site. At the molecular level, this process is thought to result from alterations in the epithelial–mesenchymal transition (EMT) mechanism of trophoblasts [9]. According to the depth of invasion, the clinical picture is classified as placenta accreta, in which chorionic villi merely adhere to the myometrium, placenta increta, in which they advance into the myometrium, and placenta percreta, in which they breach the uterine serosa and may spread to surrounding organs [8]. Because all three invasion anomalies may be present to varying degrees within the same uterus (previously termed morbidly adherent placenta…), the placenta accreta spectrum definition has been adopted since 2019. This abnormal invasion prevents spontaneous separation of the placenta during delivery, predisposing to massive hemorrhage that makes PAS one of the most challenging complications of modern obstetric practice [10].

The fundamental challenge in PAS management is achieving intraoperative hemorrhage control. Traditionally, cesarean hysterectomy is still accepted as the “gold standard” treatment, particularly in placenta percreta cases, in order to minimize the risk of massive hemorrhage and maternal mortality [11,12]. However, in addition to causing permanent loss of fertility, hysterectomy also entails serious surgical morbidities such as bladder and ureteral injuries, the need for intensive care, and massive transfusion requirements [11]. These adverse outcomes, together with patients’ desire to preserve their fertility, have led to a paradigm shift in management strategies over the past two decades [13]. Uterus-preserving (conservative) surgical methods such as the “Triple-P procedure,” leaving the placenta in situ, or local resection have gained increasing acceptance [7].

The success of uterus-preserving surgery is strictly dependent on accurate antenatal diagnosis, multidisciplinary team experience, and the center’s equipment. The aim of this study is to present our experience with a placenta previa-based cohort managed over a 13-year period at a tertiary center—within which placenta accreta spectrum was histopathologically confirmed in a defined subgroup—and, in particular, to discuss our transition to uterus-preserving surgery, the relationship of this approach with its histopathological basis, and its increasing clinical acceptability in light of the literature.

## 2. Materials and Methods

### 2.1. Study Design and Patient Population

This study is a retrospective, descriptive cohort study evaluating the operations performed by a single experienced obstetrics and gynecology specialist at the Department of Obstetrics and Gynecology, Mersin University Faculty of Medicine, over a 13-year period between January 2013 and January 2026. Approval for the study protocol was obtained from the Mersin University Clinical Research Ethics Committee (Decision No: 2026/044), and the study was conducted in accordance with the principles of the Declaration of Helsinki.

The study population consisted of 324 patients managed by the same surgeon with a diagnosis of placenta previa and/or placenta accreta spectrum and delivered by cesarean section between the specified dates. The total number of cesarean sections performed by the same surgeon during this period was recorded as 2630. Eligibility was assessed against predefined criteria. Women were included if they had completed 20 weeks of gestation, had placenta previa or a suspected placental invasion anomaly identified on antenatal ultrasonography, or received a definitive diagnosis of placenta accreta spectrum on the basis of intraoperative findings or histopathological examination, and were delivered by cesarean section by the index surgeon. Women were excluded if they underwent emergency laparotomy for a traumatic indication such as uterine rupture, or if their medical records were incomplete for the variables of interest. Because the same eligibility criteria were applied to the entire cohort and the two comparison groups were separated only after the operation had been completed, no additional group-specific inclusion criterion was applied.

### 2.2. Data Collection and Definitions

All clinical data obtained by scanning the hospital information management system and physical patient archive files were transferred to a Microsoft Excel database (Microsoft Corporation, Redmond, WA, USA). The demographic and basic obstetric characteristics of the patients included maternal age, gravidity, parity, history of abortion, number of previous cesarean sections, and mode of conception (spontaneous, in vitro fertilization [IVF], or intrauterine insemination [IUI]).

In terms of surgical and obstetric findings, gestational age and whether the operation was performed under elective or emergency conditions (with indications such as bleeding, labor pain, fetal distress, etc.) were evaluated.

Placental characteristics were classified based on ultrasonographic and intraoperative findings as the uterine location of the placenta (anterior, posterior, lateral) and the previa type. Because the cohort was accrued from 2013 onward and records were completed contemporaneously throughout the study period, the traditional four-category classification in routine clinical use at that time (total, partial, marginal, and low-lying) was retained for the entire series rather than the binary previa/low-lying terminology subsequently recommended by the 2014 Fetal Imaging Workshop. This choice preserves internal consistency across the 13 years but should be taken into account when comparing the present findings with more recent series [5]. Histopathological confirmation and depth-based grading of placenta accreta spectrum (accreta, increta, or percreta) require examination of the resected uterus and were therefore only obtainable in the 36 women who underwent peripartum hysterectomy. In women whose uterus was preserved, no specimen was available, and the operative records of the earlier years of the series did not consistently document the standardized intraoperative descriptors required for the FIGO clinical grading system. Consequently, the depth of placental invasion could not be determined for the uterus-preserving group, and the two study groups cannot be compared with respect to placenta accreta spectrum grade. Histopathological grading is accordingly reported for the hysterectomy group only, and comparisons between the groups are restricted to variables that were recorded uniformly across the entire cohort.

To evaluate maternal and neonatal outcomes, operative time, intraoperative and postoperative blood and blood product transfusion amounts (Erythrocyte Suspension [ES], Fresh Frozen Plasma [FFP], platelets, cryoprecipitate, and recombinant Factor VIIa), the requirement for a maternal intensive care unit (ICU), length of hospital stay, and reoperation rates were examined. Perioperative complications (bladder perforation, infection, wound dehiscence, intra-abdominal bleeding) were recorded. Neonatal outcomes were analyzed based on birth weight, 1st- and 5th-minute Apgar scores, and fetal mortality. To objectively assess perioperative blood loss, the difference between preoperative and postoperative hemoglobin (Hb) and hematocrit (Hct) values (ΔHb and ΔHct) was calculated.

### 2.3. Study Groups and Basis of Comparison

No a priori allocation to treatment arms was performed. The two comparison groups were defined retrospectively, according to the final surgical outcome of the index operation: the hysterectomy group comprised women in whom peripartum hysterectomy was ultimately performed (*n* = 36), and the uterus-preserving group comprised women whose uterus was left in situ at the end of the operation (*n* = 288), irrespective of whether any hemostatic adjunct had been required. Within the hysterectomy group, 32 women underwent hysterectomy after one or more uterus-preserving measures had failed to secure hemostasis, whereas in 4 women hysterectomy was performed without a preservation attempt because of the extent of invasion or hemodynamic instability recognized at laparotomy. Group membership therefore reflects the intraoperative course rather than a predefined assignment, and the comparison between the two groups should be interpreted as a description of the clinical profile associated with each outcome rather than as a comparison of two alternative treatment strategies.

### 2.4. Surgical Management and Uterus-Preserving Approaches

In all cases, a Pfannenstiel incision was used as the standard surgical approach, and the operations were performed under general anesthesia. The patients included in the study were evaluated, according to their surgical management strategies, as those who underwent peripartum hysterectomy and those managed with uterus-preserving (conservative) surgical methods. Uterus-preserving surgical approaches were applied alone or in combination, depending on the severity of the patient’s intraoperative bleeding, the depth of placental invasion, and the surgeon’s clinical assessment. All surgical consumables used in the uterus-preserving procedures (Foley catheters, silicone intrauterine balloons, absorbable polyglactin 910 sutures, and sterile packing gauze), as well as the ultrasound systems used for antenatal evaluation, were those routinely supplied to the department through institutional procurement. Because the cohort spans 13 years, the manufacturers of these standard, interchangeable items varied over time and were not recorded in the operative notes; no product-specific or proprietary device was required for any step of the algorithm.

**Figure 1:**
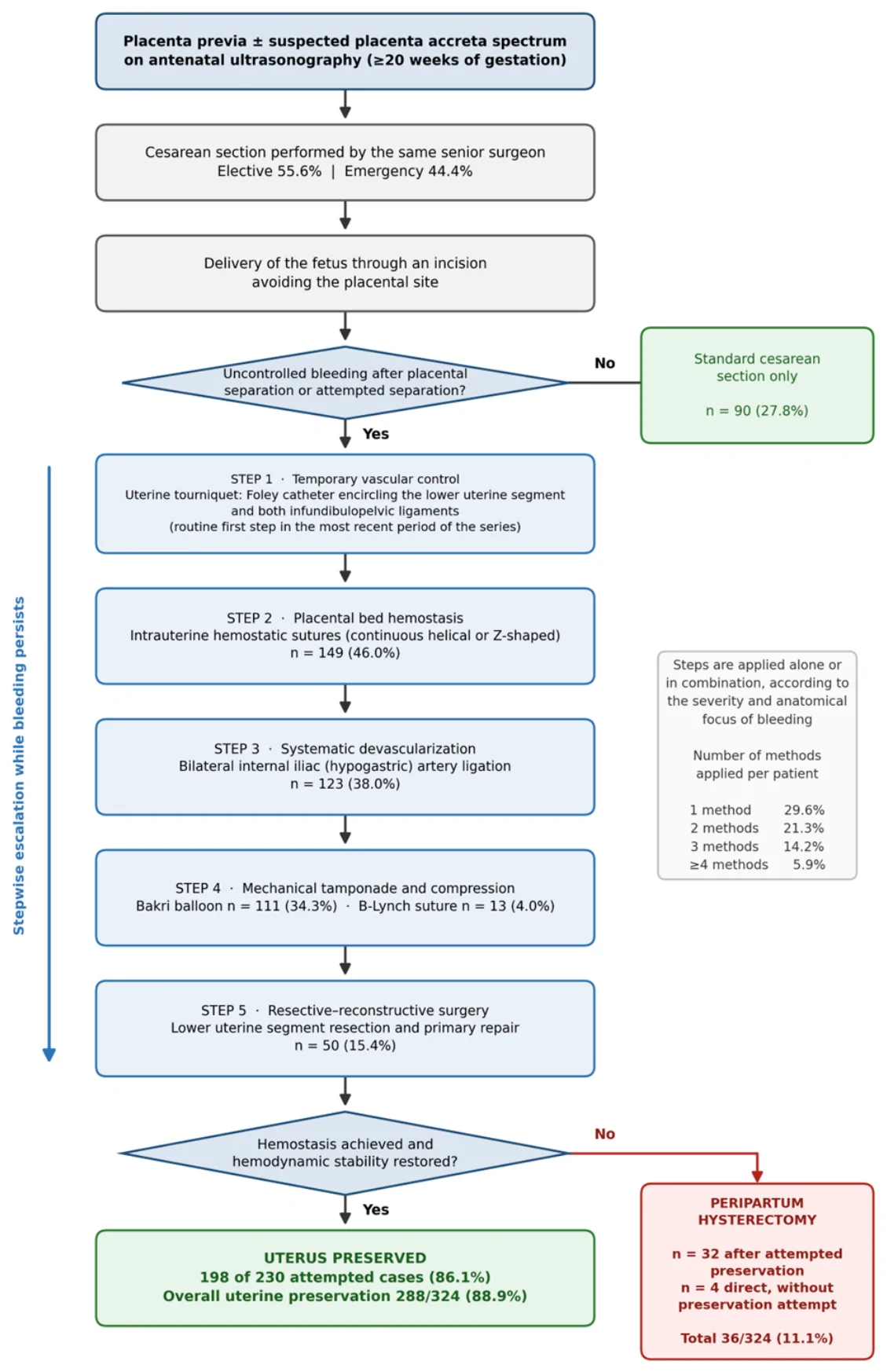
Step-up uterus-preserving surgical algorithm for placenta previa and placenta accreta spectrum (*n* = 324). **Footnotes:** The algorithm was reconstructed retrospectively from the operative records of all 324 cases and reflects the escalation pattern consistently followed by the operating surgeon; it was not applied as a prospectively written institutional protocol. Steps were not mutually exclusive and were applied alone or in combination according to the severity and anatomical focus of bleeding; consequently, the percentages of individual methods do not sum to 100%. All percentages are calculated relative to the total cohort (*n* = 324), except for the uterine preservation rate among attempted cases (198/230), which is calculated relative to the patients in whom at least one uterus-preserving method was attempted. PAS: placenta accreta spectrum.

Although the series was managed retrospectively and no written institutional protocol existed at its outset, the surgical decision-making of the operating surgeon followed a consistent and reproducible escalation pattern that could be reconstructed from the operative records of all 324 cases. This pattern, hereafter referred to as the step-up approach, is summarized in Figure 1. Hemostatic interventions were escalated stepwise—from temporary vascular control, through placental bed suturing and systematic devascularization, to mechanical tamponade and, finally, resective–reconstructive surgery—and peripartum hysterectomy was reserved for cases in which bleeding remained uncontrolled despite this escalation. It should be emphasized that this algorithm represents the retrospectively reconstructed pattern of a single surgeon’s practice rather than a prospectively applied protocol. The individual steps are defined below.

**Figure 2:**
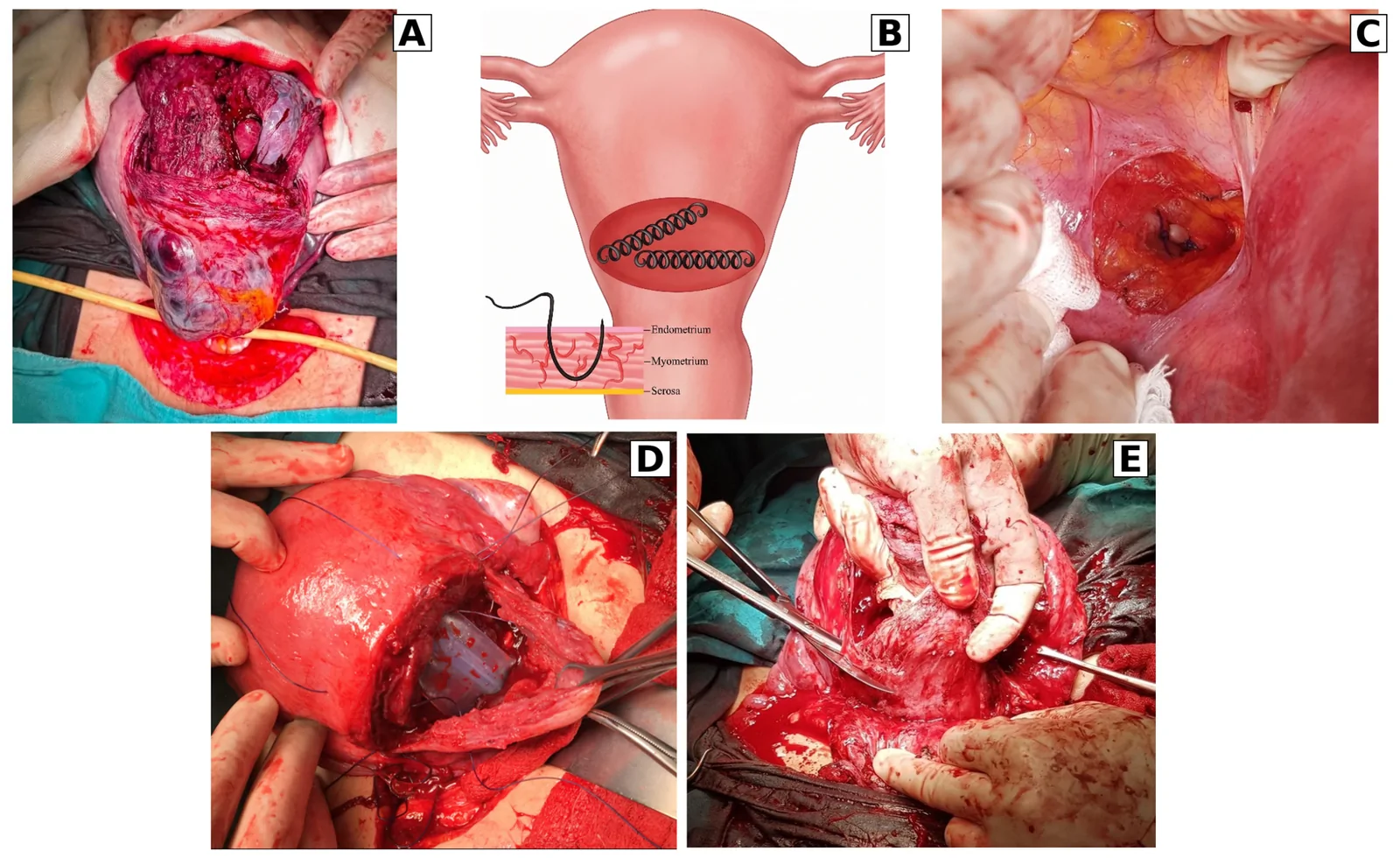
Uterus-preserving surgical techniques. **Footnotes:** (**A**) Uterine Tourniquet. (**B**) Intrauterine hemostatic (helical) sutures. (**C**) Internal iliac artery ligation (IIAL). (**D**) B-Lynch suture and Bakri balloon tamponade (combined use). (**E**) Uterine lower segment resection. PAS: placenta accreta spectrum; IIAL: internal iliac artery ligation.

**Uterine Tourniquet (Temporary Vascular Control):** Immediately after delivery of the fetus and prior to any attempt at placental separation, a Foley catheter tourniquet was encircled around the lower uterine segment, bilaterally incorporating the infundibulopelvic ligaments, and tightened. This provided temporary, fully reversible mechanical occlusion of uterine inflow, markedly reducing intraoperative blood loss during placental delivery, resection, and reconstruction. In the most recent period of the series, this maneuver has been adopted as a routine first step in all PAS cases (Figure 2A).

**Intrauterine Hemostatic Sutures:** Continuous helical or Z-shaped sutures applied to control active bleeding from the placental bed; No. 0 or 1 absorbable suture material was used (Figure 2B).

**Internal Iliac Artery Ligation (IIAL):** After retroperitoneal dissection, ligation of the bilateral internal iliac (hypogastric) artery with an absorbable suture 2–3 cm distal to the bifurcation of the common iliac artery; this reduces uterine perfusion pressure and controls collateral circulation in the distal vascular network to a level that allows hemostasis (Figure 2C).

**Bakri Balloon Tamponade:** Application of hydrostatic pressure on the placental bed and uterine lower segment by inflating a silicone Bakri balloon, placed into the uterine cavity through the cesarean incision, with sterile saline (180–240 mL). The balloon was controlled and removed after being monitored for approximately 24 h postoperatively via the guide tube (Figure 2D).

**B-Lynch Suture (Uterine Compression Suture):** A longitudinal compression suture technique performed with No. 1 absorbable suture material (polyglactin 910), starting from both uterine cornua and wrapping the uterine fundus from the anterior and posterior surfaces, compressing the uterus along its long axis. The suture also encompasses the edges of the lower segment cesarean incision at both uterine cornua (Figure 2D).

**Uterine Lower Segment Resection:** In cases where the placenta had invaded up to the serosa and no tissue remained for repair once the placenta was removed, excision of the lower segment tissues down to healthy tissue near the internal cervical os and primary suture repair of the uterine defect (Figure 2E).

**Uterine Packing:** In diffuse placental bed bleeding that responds inadequately to conventional hemostatic methods, providing mechanical pressure and hemostasis by placing sterile packing gauze into the uterine cavity; the packing was removed in a controlled manner at the postoperative 24th–48th hour.

The demographic and surgical data of patients who underwent these methods were comparatively analyzed against those who proceeded directly to hysterectomy or were managed with a standard cesarean section without requiring any additional hemostatic intervention.

### 2.5. Statistical Analysis

Statistical analyses were performed using IBM SPSS Statistics for Windows, version 23.0 (IBM Corp., Armonk, NY, USA). Normality was assessed with the Kolmogorov–Smirnov test. Continuous variables are presented as median with interquartile range (IQR; 25th--75th percentile), and categorical variables as number (*n*) and percentage (%). Two independent groups were compared with the Mann–Whitney U test for continuous variables and with the chi-square or Fisher’s exact test for categorical variables; the Wilcoxon signed-rank test was used for paired hemoglobin comparisons, and monotonic associations between ordinal and continuous variables were assessed with Spearman’s rank correlation coefficient.

Factors independently associated with failure of uterine preservation were examined by binary logistic regression, with peripartum hysterectomy as the dependent variable. Candidate predictors were confined to preoperative variables recorded uniformly throughout the period: maternal age, number of previous cesarean sections, gestational age, placental location, previa type, emergency versus elective surgery, and surgical period as a surrogate for accumulated experience. Intraoperative and postoperative variables were excluded as consequences rather than determinants of the outcome, and placenta accreta spectrum grade could not be entered because histopathological grading was available only in women who underwent hysterectomy. Variables with *p* < 0.10 on univariable analysis were considered for the multivariable model; because more variables met this threshold than the events-per-variable principle permitted with the 36 available events, the three showing the strongest univariable association were retained. Results are reported as odds ratios (OR) with 95% confidence intervals (CI); discrimination and calibration were assessed by the area under the receiver operating characteristic curve and the Hosmer–Lemeshow test, and internal validity by bootstrap resampling (1000 replications) with calculation of the optimism-corrected area under the curve and the calibration slope. Where complete separation precluded maximum-likelihood estimation, Firth’s penalized likelihood was used as a sensitivity analysis. A two-sided *p* < 0.05 was considered significant.

## 3. Results

Between 1 January 2013 and 1 January 2026, 324 women with placenta previa were delivered by cesarean section by the same senior surgeon; these accounted for 12.3% of the 2630 cesarean sections performed by this surgeon over the same period. The demographic, obstetric, and neonatal characteristics of the cohort are presented in Table 1. Almost all pregnancies were conceived spontaneously, and slightly more than half of the operations were elective. One neonatal death occurred, and no maternal death was recorded.

**Figure 3:**
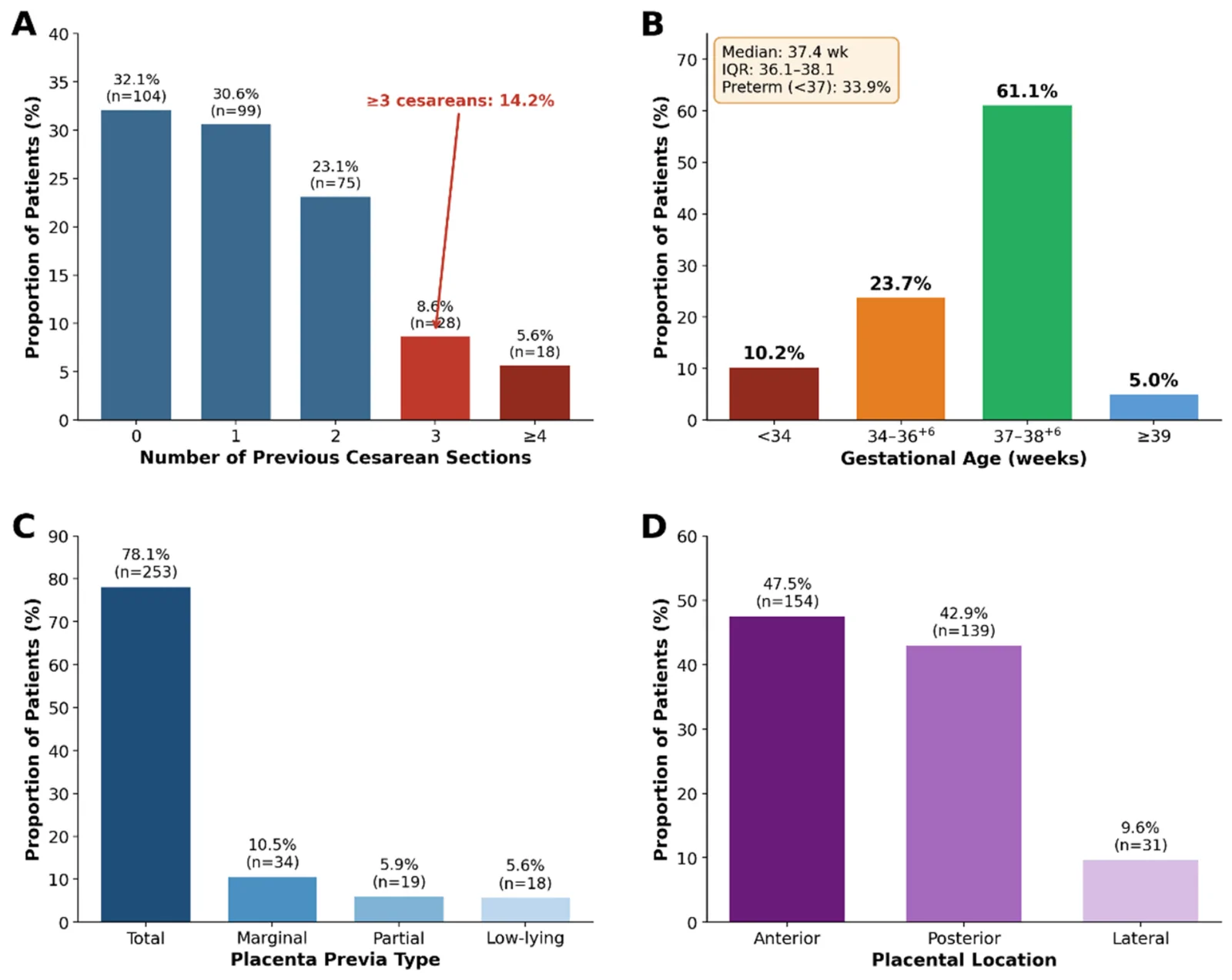
Patient Characteristics (*n* = 324). **Footnotes:** Characteristics of patients who underwent cesarean section for placenta previa. (**A**) Distribution of the number of previous cesarean sections. (**B**) Distribution of gestational age at delivery. (**C**) Distribution of placenta previa type. (**D**) Placenta previa location.

The distribution of the number of previous cesarean sections, of gestational age at delivery, and of the type and location of placenta previa is shown in Figure 3. Two thirds of the women had at least one previous cesarean section, one third delivered before 37 weeks of gestation, and total previa was by far the most frequent type (78.1%). Anterior and posterior placental locations occurred in approximately equal proportions, and a lateral location was uncommon.

Preoperative tocolysis was not applied in 93.2% of patients (*n* = 302), while it was applied in 6.8% (*n* = 22) for the purpose of administering antenatal corticosteroids due to preterm labor. Among the emergency cases, the most frequent indications were vaginal bleeding (22.8%, *n* = 74) and labor pain (11.1%, *n* = 36), while the coexistence of labor pain and bleeding was observed in 4.0% (*n* = 13). Acute fetal distress, oligohydramnios, intrauterine growth restriction, or coexistence of fetal demise constituted an indication in 3.4% (*n* = 11). Premature rupture of membranes (PROM) was observed in 1.2% (*n* = 4). The rate of combined indication cases, in which more than one emergency indication coexisted, was found to be 1.9% (*n* = 6).

**Figure 4:**
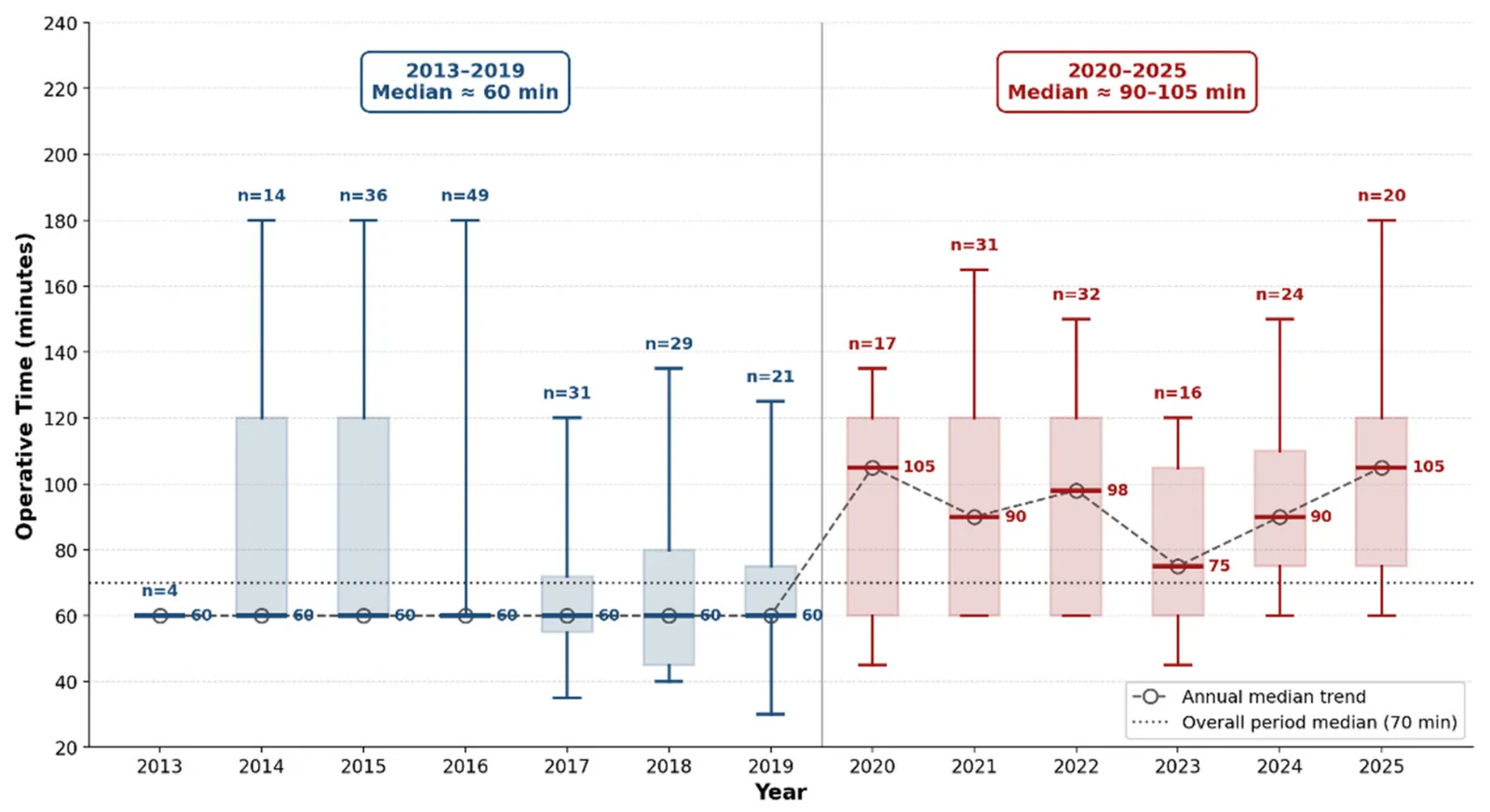
Distribution of Operative Times by Year (Box Plot), 2013–2025 (*n* = 324). **Footnotes:** The boxes show the interquartile range (IQR, Q1–Q3), the line within the box shows the median, and the whiskers show the minimum and maximum values. Blue boxes represent the 2013–2019 period and red boxes represent the 2020–2025 period. The gray dashed line shows the annual median trend, and the dotted horizontal line shows the overall median of the 13-year period (70 min). The lengthening of operative time from 2020 onward reflects the progressively wider and more frequently combined use of uterus-preserving maneuvers, rather than any change in operating theater conditions or surgical team.

The median operative time for all patients was 70 min (IQR: 60–120), with the duration ranging from 30 to 180 min. When operative times by year were examined, the median time remained stable at around 60 min during the 2013–2019 period (combined median for 2013–2019: 60 min, IQR: 60–80), while a marked increasing trend was observed from 2020 onward, with the median time rising to 90 min (IQR: 75–120) in the 2020–2025 period. The longest median durations reached 105 min (IQR: 75–120) in 2020 and 2025 (Figure 4). This increase paralleled the wider adoption of uterus-preserving surgery rather than any change in operating theater conditions, surgical team, or anesthetic practice. The proportion of operations in which at least one uterus-preserving maneuver was performed rose from 59.2% in 2013–2019 to 86.4% in 2020–2025, and the mean number of maneuvers per operation increased from 1.0 to 1.9 (*p* < 0.001). Operative time rose in parallel with the number of maneuvers applied (median 60 min when none or one was used, 90 min with two, and 120 min with four; Spearman ρ = 0.54, *p* < 0.001), whereas among operations requiring no additional maneuver the median operative time was identical in the two periods (60 min in both).

**Figure 5:**
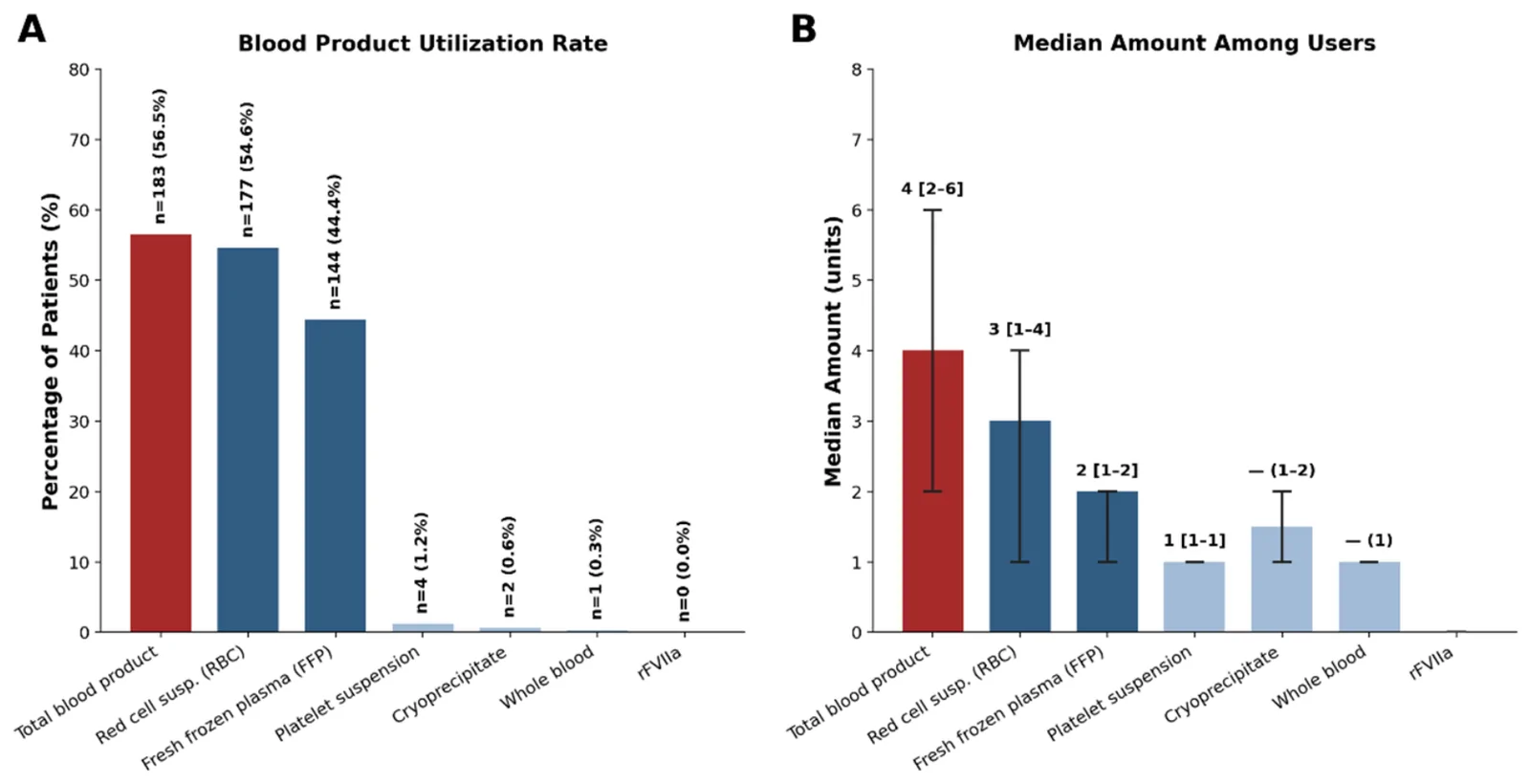
Perioperative Blood Product Use (*n* = 324). **Footnotes:** (**A**) Number of patients (*n*) receiving each blood product and the rate of use (%) relative to the total number of patients (*n* = 324). (**B**) Median number of units transfused among patients who received the respective blood product, shown as median [interquartile range]; the square brackets denote the interquartile range and are not reference citations. For cryoprecipitate and whole blood, the number of users was too small for a meaningful median, and the observed values are given in parentheses.

At least one blood product transfusion was administered to 56.5% of patients (*n* = 183) during the perioperative period. Erythrocyte suspension (ES) was the most frequently used blood product, transfused to 54.6% of patients (*n* = 177); among those who received it, the median amount was three units (IQR: 1–4). Fresh frozen plasma (FFP) was administered to 44.4% of patients (*n* = 144) (median two units, IQR: 1–2). Platelet suspension (*n* = 4, 1.2%), cryoprecipitate (*n* = 2, 0.6%), and whole blood (*n* = 1, 0.3%) were used in only a limited number of cases; recombinant activated factor VIIa (rFVIIa) was not used in any case. The median total transfusion in patients who received blood products was calculated as 4 units (IQR: 2–6, maximum 29 units) (Figure 5).

The proportion of patients requiring a postoperative intensive care unit (ICU) was found to be 8.0% (*n* = 26). The median length of stay for patients admitted to the ICU was 1 day (IQR: 1–2, maximum 5 days). The median postoperative hospital stay was 2 days (IQR: 2–3, range 0–11 days), and only six women (1.9%) stayed for 7 days or longer. The number of cases requiring reoperation was four (1.2%); of these, two cases involved repair of intra-abdominal bleeding and/or urinary system injury, one case involved a postoperative infection/ARDS picture, and in one case no specific indication was noted in the record file (Table 2).

**Figure 6:**
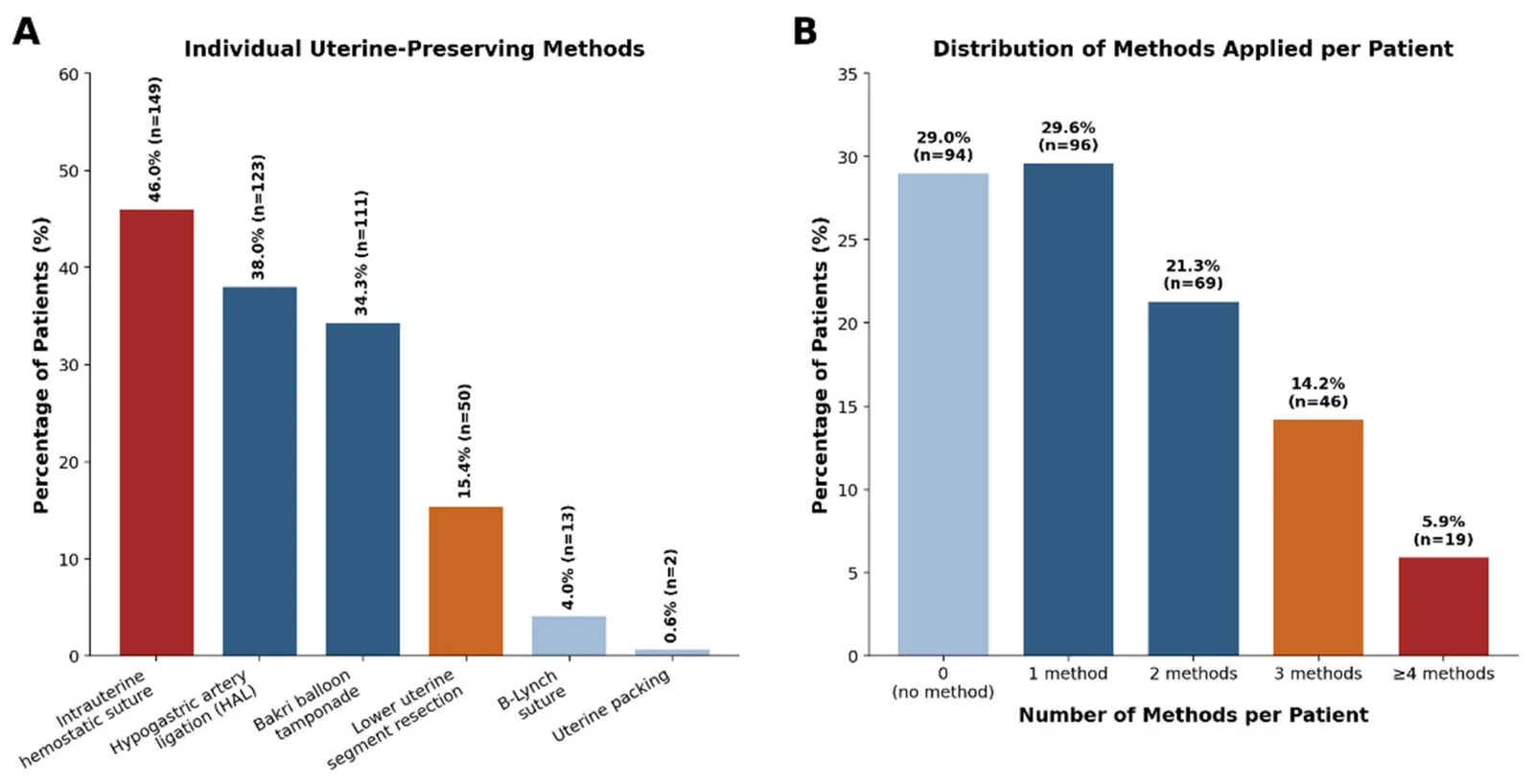
Uterus-Preserving Surgical Methods (*n* = 324). **Footnotes:** (**A**) Frequency of use of individual methods (number and proportion of patients receiving each method; the methods are not mutually exclusive, and more than one method may have been applied in a single patient). (**B**) Distribution of the total number of uterus-preserving methods applied per patient (0 = including cases in which no method was applied). See Supplementary Table S1 for the full distribution of combinations. IHS: intrauterine hemostatic suture; IIAL: hypogastric artery ligation.

At least one uterus-preserving surgical method was applied to 71.0% of patients (*n* = 230) during the perioperative period; in the remaining 29.0% (*n* = 94), no uterus-preserving method was applied—of these, 90 patients were managed with standard cesarean section alone, while 4 underwent direct peripartum hysterectomy without any preserving attempt. Of the 230 patients in whom a uterus-preserving method was attempted, the uterus was successfully preserved in 198, whereas in 32 the bleeding could not be controlled and peripartum hysterectomy was required. Together with the 90 patients managed by standard cesarean section, the uterus was preserved in 288 patients (88.9%) overall; peripartum hysterectomy was performed in a total of 36 patients (11.1%), comprising 32 cases following an attempted preservation and four direct hysterectomies. The frequency of use of the individual methods is shown in Figure 6A; the intrauterine hemostatic suture was the most frequently applied, followed by internal iliac artery ligation and Bakri balloon tamponade.

The number of methods applied per patient is shown in Figure 6B, ranging from a single method in 96 women (29.6%) to four or more in 19 (5.9%). The most frequent single intervention was the intrauterine hemostatic suture and the most frequent combination was the Bakri balloon together with the intrauterine hemostatic suture; the full distribution of combinations is presented in Supplementary Table S1.

**Figure 7:**
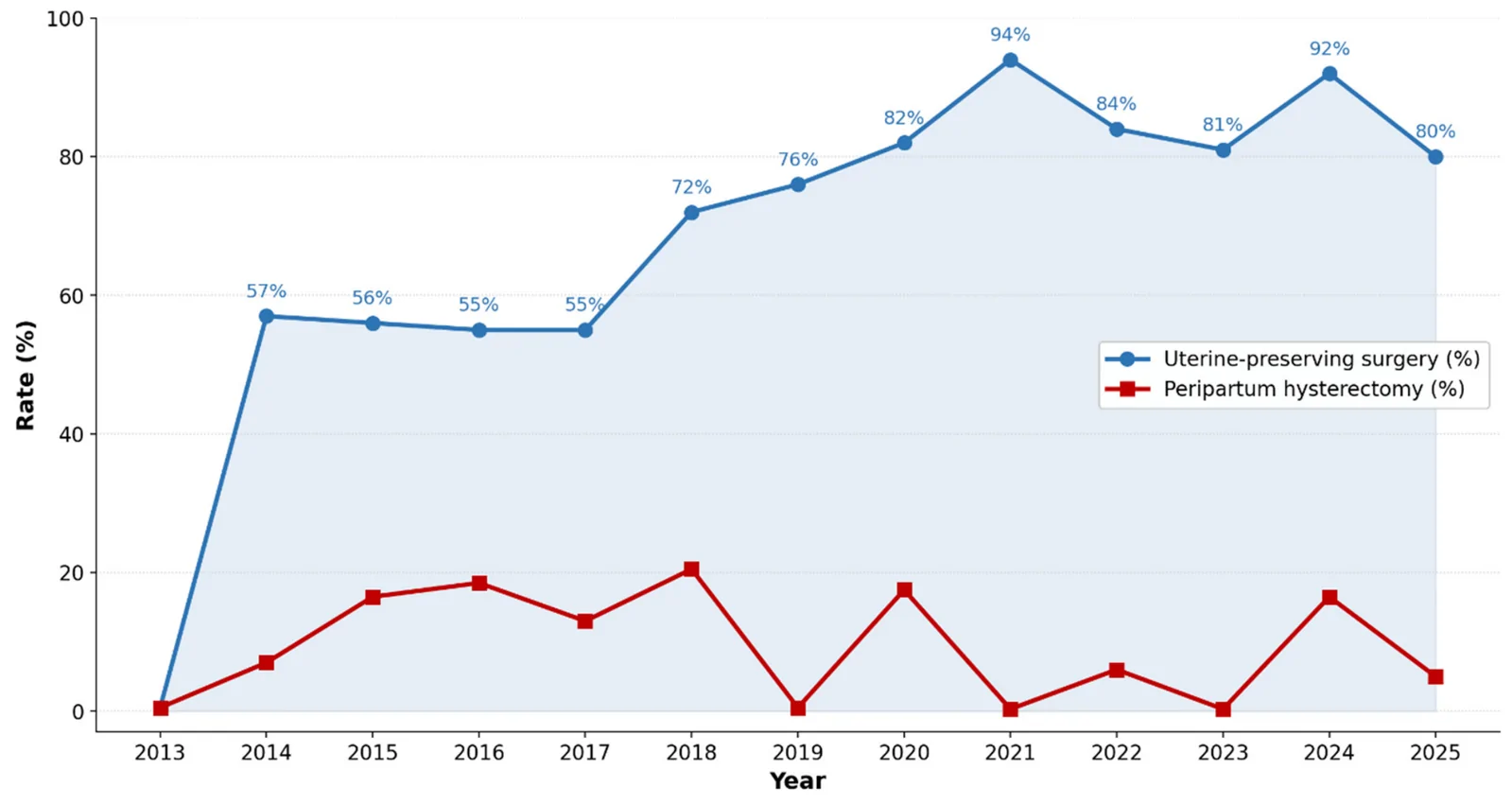
Uterus-Preserving Surgery and Peripartum Hysterectomy Rates by Year (%). **Footnotes:** The blue line shows the annual rate of uterus-preserving surgery (at least one method), and the red line shows the annual peripartum hysterectomy rate. Percentages were calculated relative to the total number of previa cases in each year.

When the rate of uterus-preserving surgery use (i.e., the proportion of cases in which at least one uterus-preserving method was attempted) by year was evaluated, no uterus-preserving method was applied in any case in 2013 (0%), the rate remained relatively stable at 55–57% during the 2014–2017 period, and showed a marked increasing trend from 2018 onward. The uterus-preserving surgery rate reached 93.5% in 2021 and 91.7% in 2024, consistently remaining at or above 80% in the final period of the study. During the same period, the peripartum hysterectomy rate followed a relatively variable course; no hysterectomy was performed in 2019, 2021, and 2023, while the highest rate was observed in 2018 (20.7%). These findings reflect the gradual and marked adoption of uterus-preserving approaches in the final period of the study (Figure 7).

**Figure 8:**
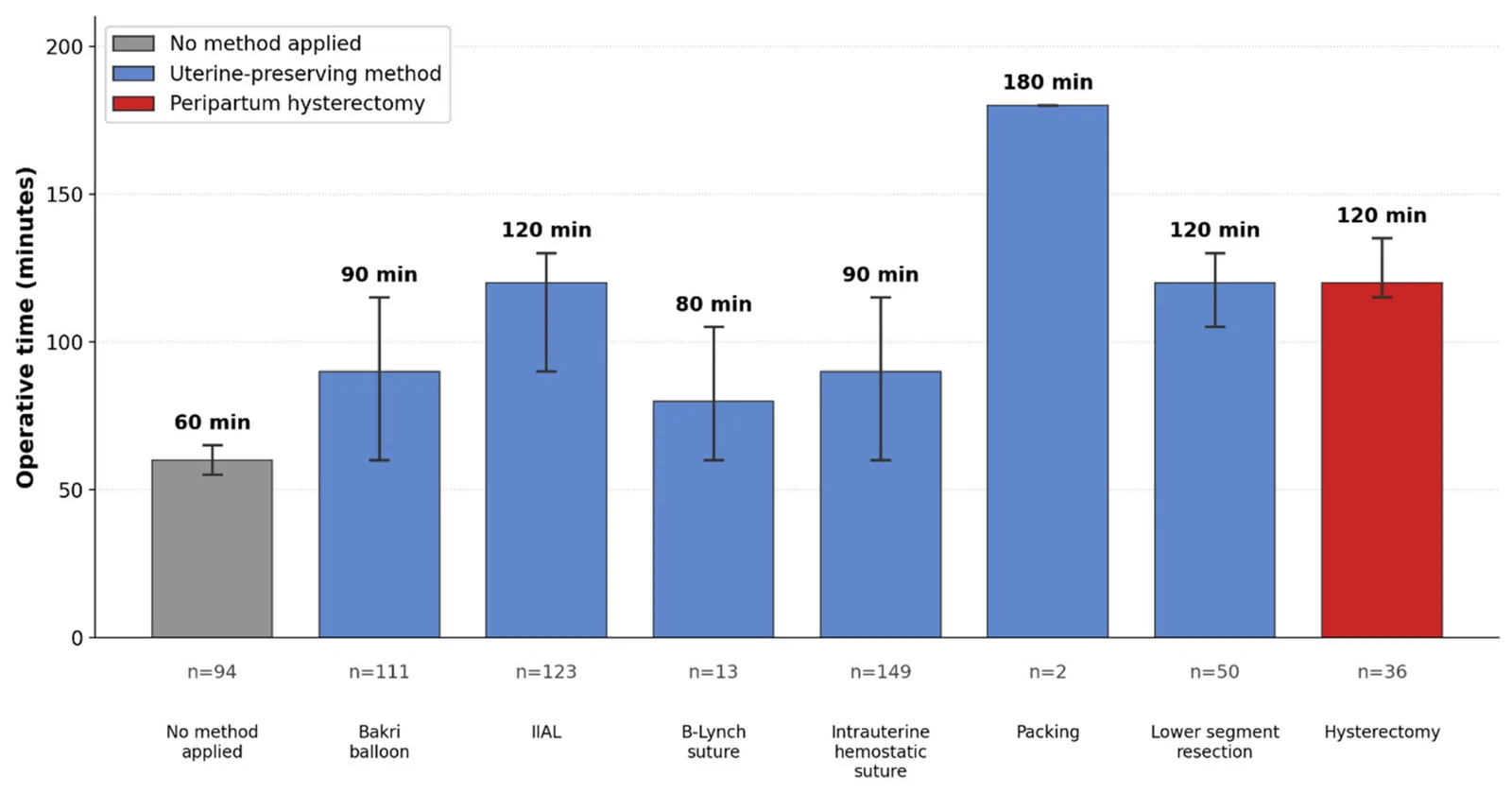
Distribution of Operative Time by Type of Surgery Performed (Median and IQR). **Footnotes:** The bars show the median operative time (minutes) in the respective group, and the error bars show the interquartile range (IQR: Q1–Q3). Patient grouping is not mutually exclusive; when more than one method was applied in a patient, they were counted in each respective group. The “No method applied” group includes cases in which no uterus-preserving hemostatic method was used (*n* = 94, regardless of whether hysterectomy was performed); patients who underwent hysterectomy are additionally shown in the separate “Hysterectomy” group. The reconciliation of group sizes across the figures using method-based groupings is presented in Figure S1. IQR: interquartile range; IIAL: internal iliac artery ligation.

When operative times were examined by the type of surgery performed, the median operative time remained stable at around 60 min in cases in which no uterus-preserving method was applied, while it was markedly prolonged in cases in which a preserving method was applied (median 80–120 min). The longest operative times were observed in the IIAL (median 120 min, IQR: 90–120), lower segment resection (median 120 min, IQR: 105–120), and peripartum hysterectomy (median 120 min, IQR: 116–135) groups. This finding demonstrates that, in complex and hemorrhagic cases, both vascular control and surgical reconstruction require additional time, and that the uterus-preserving approach imposes a surgical burden close to that of hysterectomy (Figure 8).

During the study, peripartum hysterectomy was performed in 36 patients (11.1%); in 288 patients (88.9%), hysterectomy was avoided and a uterus-preserving approach was adopted. As a result of pathological examination in the cases that underwent hysterectomy, diagnoses of placenta accreta (30.6%, *n* = 11), placenta increta (30.6%, *n* = 11), and placenta percreta (13.9%, *n* = 5) were confirmed, and 75.0% of cases (*n* = 27) were histopathologically classified as placenta accreta spectrum (Table 3).

When the hysterectomy and non-hysterectomy groups were compared clinically, the median age (35 vs. 32 years; *p* = 0.008), operative time (120 vs. 60 min; *p* < 0.001), total blood product use (6 vs. 1 units; *p* < 0.001), and postoperative hospital stay (3 vs. 2 days; *p* < 0.001) were significantly higher in the hysterectomy group. No significant difference was found between the two groups in terms of gestational age (37.6 vs. 37.3 weeks; *p* = 0.932) (Table 3).

Factors associated with failure of uterine preservation were examined by logistic regression (Table 4). On univariable analysis, maternal age and the number of previous cesarean sections were associated with peripartum hysterectomy, whereas gestational age and emergency admission to surgery were not. The relationship with prior cesarean delivery was graded: hysterectomy was required in 1.0% of women with no previous cesarean section, 7.1% with one, 17.3% with two, and 32.6% with three or more. All 36 hysterectomies occurred in women with total previa and none among the 71 women with partial, marginal, or low-lying previa. In the multivariable model, each additional previous cesarean section more than doubled the odds of hysterectomy (OR 2.41, 95% CI 1.72–3.36; *p* < 0.001), whereas operation during the later period of the series was associated with a substantially lower risk (OR 0.24, 95% CI 0.10–0.59; *p* = 0.002), the crude hysterectomy rate falling from 14.1% in 2013–2019 to 7.1% in 2020–2025. Maternal age retained borderline significance (*p* = 0.057). Discrimination was good and calibration adequate, and a penalized (Firth) sensitivity analysis that allowed total previa to be retained in the model left the estimates for previous cesarean sections and surgical period essentially unchanged.

**Figure 9:**
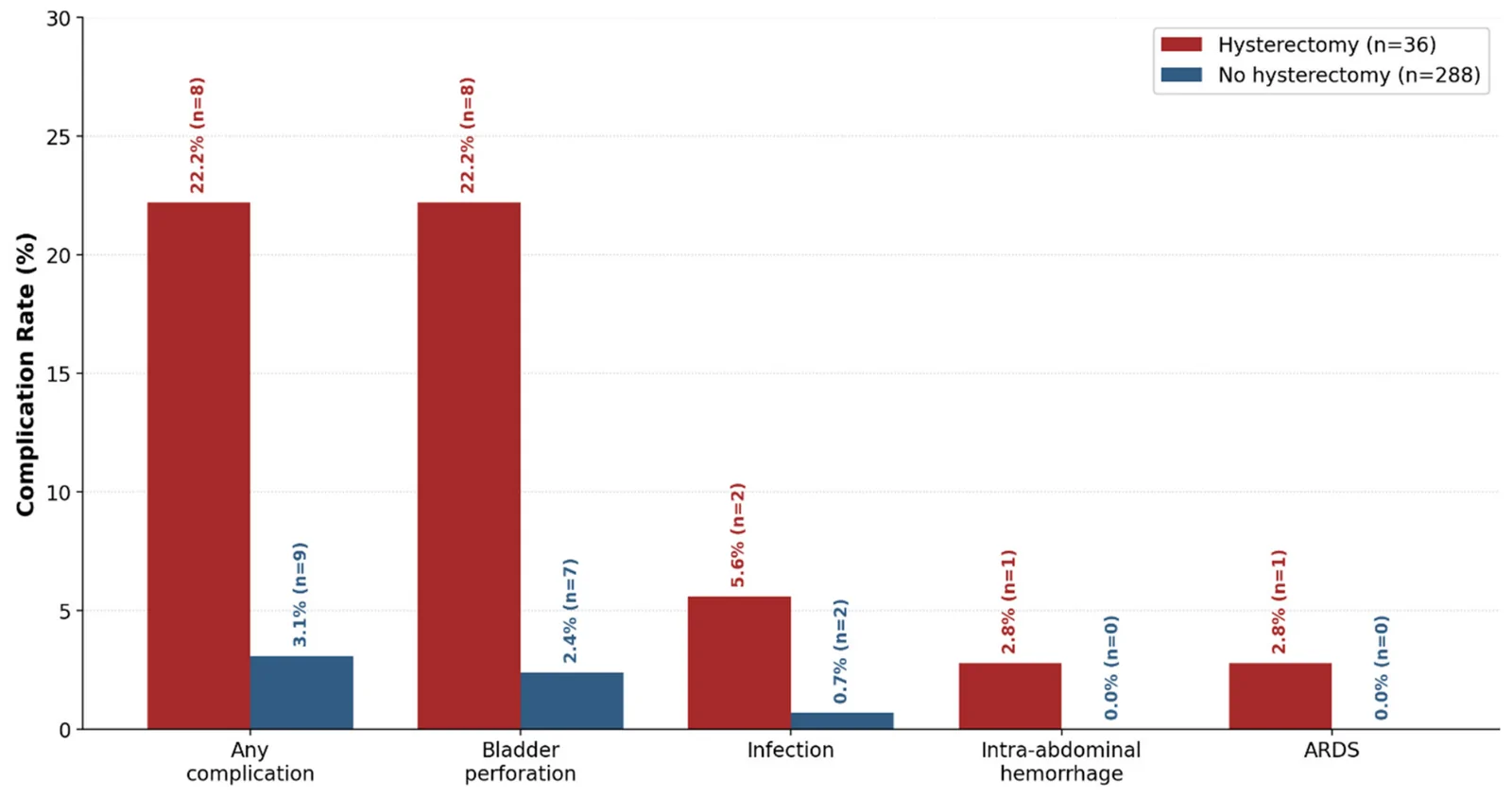
Distribution of Perioperative Complications by Hysterectomy Status (*n* = 324). **Footnotes:** The rate of occurrence (%) and absolute number of cases (*n*) of each complication type in the hysterectomy (*n* = 36, red) and non-hysterectomy (*n* = 288, blue) groups are shown. ARDS: acute respiratory distress syndrome.

During the study, at least one perioperative complication developed in 5.2% of patients (*n* = 17); no complication was observed in 94.8% (*n* = 307). The most frequent complication was bladder perforation, found in 15 patients (4.6%). Other complications included infection (1.2%, *n* = 4), intra-abdominal bleeding (0.3%, *n* = 1), and acute respiratory distress syndrome (ARDS; 0.3%, *n* = 1). Gastrointestinal system injury, wound dehiscence, and maternal mortality were not observed in any case during the 13-year study (Figure 9).

When complications were evaluated according to hysterectomy status, the rate of developing any complication in the hysterectomy group was 22.2% (*n* = 8), approximately sevenfold higher than in the non-hysterectomy group (3.1%, *n* = 9). Bladder perforation was also markedly more frequent in the hysterectomy group (22.2%, *n* = 8 vs. 2.4%, *n* = 7). Infection (5.6% vs. 0.7%), intra-abdominal bleeding (2.8% vs. 0.0%), and ARDS (2.8% vs. 0.0%) were also observed at higher rates in the hysterectomy group. These findings support that hysterectomy is strongly associated with serious maternal morbidity and underscore the importance of preferring uterus-preserving methods first (Figure 9).

**Figure 10:**
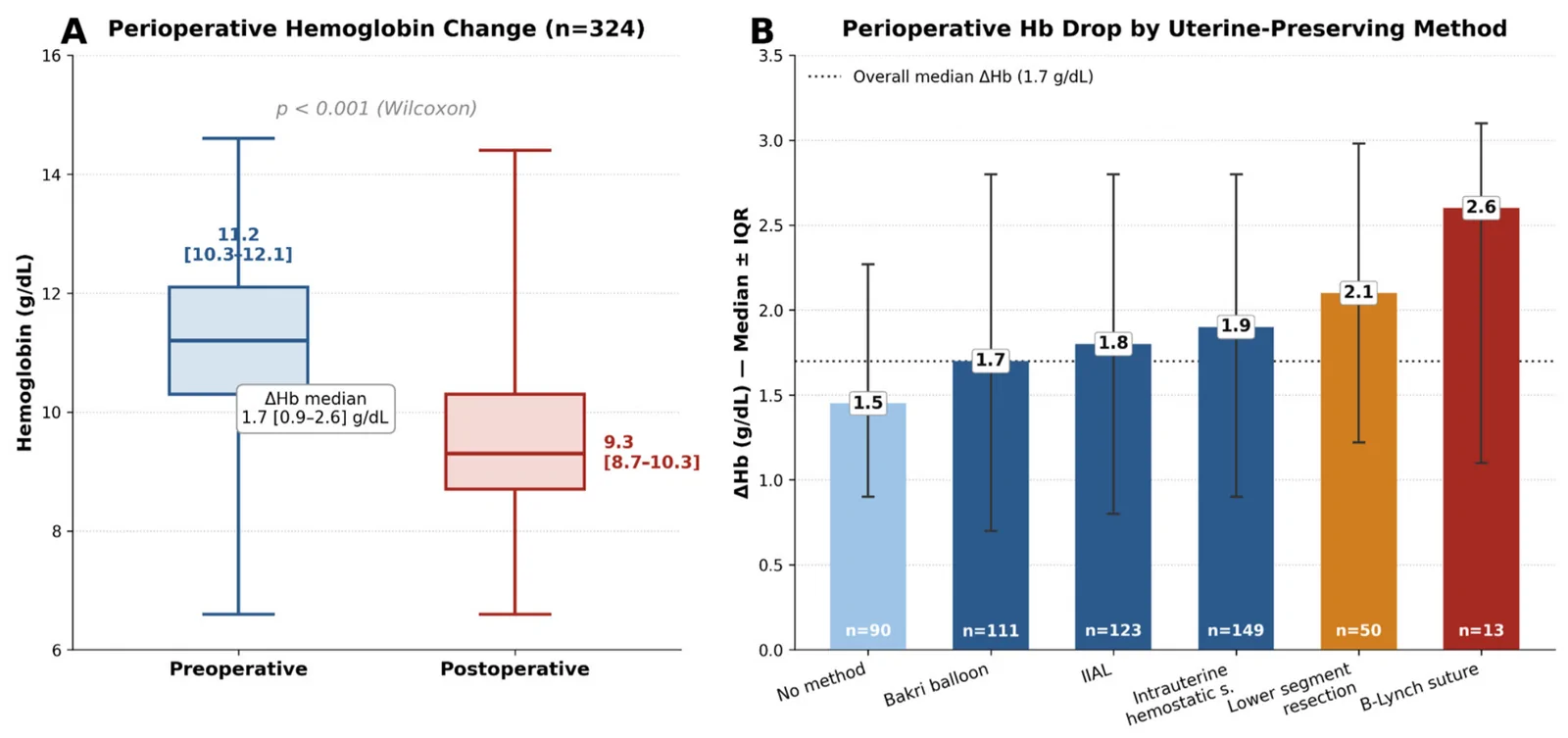
Perioperative Hemoglobin Change (*n* = 324). **Footnotes:** (**A**) The median and interquartile range (IQR, box) and minimum–maximum (whisker) distributions of preoperative and postoperative hemoglobin (Hb) values. The arrow indicates the perioperative Hb decrease (ΔHb); the difference between preoperative and postoperative Hb values is statistically significant (Wilcoxon signed-rank test, *p* < 0.001). (**B**) Distribution of ΔHb by uterus-preserving surgical method; the bars show the median and the error bars show the interquartile range (IQR: Q1–Q3). The dotted horizontal line shows the overall ΔHb median for all patients (1.7 g/dL). Because the methods are not mutually exclusive, a patient receiving more than one method is counted in each respective group; the “No method” group (*n* = 90) comprises cases in which neither a uterus-preserving method nor hysterectomy was performed. IHS: intrauterine hemostatic suture; IIAL: hypogastric artery ligation.

Perioperative complete blood count values were analyzed in all 324 patients. The preoperative median hemoglobin (Hb) value was 11.2 g/dL (IQR: 10.3–12.1), and the preoperative median hematocrit was 33.5% (IQR: 31.0–36.0). In the postoperative period, the median Hb value declined to 9.3 g/dL (IQR: 8.7–10.3) and the median hematocrit fell to 28.0% (IQR: 26.0–30.1). The perioperative Hb decrease (ΔHb) was calculated as a median of 1.7 g/dL (IQR: 0.9–2.6), and the hematocrit decrease as a median of 5.1% (IQR: 2.5–8.0). The difference between preoperative and postoperative Hb values was statistically significant (Wilcoxon signed-rank test, *p* < 0.001) (Figure 10A).

When ΔHb values were compared across the uterus-preserving method groups, the highest Hb decrease was observed in the B-Lynch suture group (median 2.6 g/dL, IQR: 1.1–3.1; *n* = 13) and the uterine lower segment resection group (median 2.1 g/dL, IQR: 1.2–3.0; *n* = 50). These were followed by intrauterine hemostatic suture (median 1.9 g/dL; *n* = 149), IIAL (1.8 g/dL; *n* = 123), and Bakri balloon tamponade (1.7 g/dL; *n* = 111). These findings indicate that the B-Lynch suture and lower segment resection were preferred in more complex and severely hemorrhagic cases (Figure 10B).

## 4. Discussion

In this study, 324 PAS/placenta previa cases managed by a single senior surgeon over an uninterrupted 13-year period were presented; the results achieved—zero maternal mortality, an 88.9% uterus-preservation rate, and an 11.1% peripartum hysterectomy rate—reflect the clinical limit attainable in the management of this high-risk obstetric condition through a standardized surgical discipline. Current FIGO consensus data reveal that the prevalence of placenta accreta spectrum (PAS) has increased approximately tenfold over the past two decades, reaching a level of 1:588, owing to the global rise in cesarean sections [14,15]. Nevertheless, in large multicenter series, inter-operator technical variations arising from different surgical teams complicate management outcomes [16]. The present series, in which all cases were managed by a single surgeon, constitutes one of the rare long-term datasets that completely eliminates this methodological limitation and documents how surgical standardization and anatomical familiarity were refined over time [17].

The term step-up requires explicit definition, since it describes here a pattern of surgical behavior rather than a formally published protocol. In this series it denotes a fixed hierarchy of hemostatic interventions in which the least invasive and most readily reversible measure is applied first, and each subsequent measure is added only if bleeding persists: temporary vascular control with a uterine tourniquet, followed by placental bed suturing, systematic devascularization by internal iliac artery ligation, mechanical tamponade or compression, and finally resective–reconstructive surgery of the lower uterine segment, with peripartum hysterectomy reserved as the terminal option (Figure 1). Three features distinguish this from an ad hoc combination of techniques: the order of escalation was invariant, the decision to escalate was governed by a single criterion—persistence of clinically significant bleeding after the preceding step—and hysterectomy was never undertaken while an unused step remained available. The approach, however, was not prospectively codified. It emerged progressively during the early years of the series and was applied in its complete form only in the later period, a development that is itself reflected in the temporal changes reported here.

The demographic profile of the cohort is largely consistent with the established risk factors for PAS. The median age was 32 years, and 14.2% of cases had a history of three or more previous cesarean sections. In the presence of placenta previa, the risk of PAS increases non-linearly with the number of cesarean sections; for the first, second, and third repeat cesarean sections, this risk rises to approximately 3%, 11%, and 40%, respectively [4]. In our series, the total previa rate of 78.1% and anterior location of 47.5% indicate that the pathophysiology of supra-scar anterior placentation—which increases the depth of invasion and markedly raises the risk of surgical complications—was predominant in this cohort [18,19]. Kirovakov et al. emphasized that inadequate decidualization in the lower segment and the presence of scar tissue deepen trophoblastic invasion, and that anterior total previa cases constitute the most severe clinical form of this pathophysiology [20]. The presence of assisted reproductive technology (ART) use in 4.6% of cases is also a finding consistent with the abnormal placentation risk profile in the literature [6].

The most notable temporal finding of the series is that the uterus-preserving surgery rate, which was zero in 2013, systematically rose above 90% in 2021 and thereafter. In parallel, the peripartum hysterectomy rate followed a variable course, with no hysterectomy performed in 2019, 2021, and 2023. This temporal evolution concretely demonstrates how a single surgical team can safely transition from radical approaches to organ-preserving strategies as it gains cumulative experience. Importantly, this shift was not explained by a more favorable case mix in the later years: after adjustment for maternal age and the number of previous cesarean sections, operation during the 2020–2025 period remained independently associated with a markedly lower probability of peripartum hysterectomy (OR 0.24, 95% CI 0.10–0.59; *p* = 0.002; Table 4). The number of previous cesarean sections was the strongest individual determinant of failure of uterine preservation, each additional prior section more than doubling the odds of hysterectomy. A similar positive learning curve was also observed in the 16-year, 308-case Turkish tertiary-center series of Cetinkaya Demir et al.; a marked decrease in major morbidity rates over the years was reported [19]. In the multicenter IS-PAS database of van Beekhuizen et al., a temporal decline in median blood loss and complication rates was also reported, and this finding was attributed to a positive learning curve [16]. As Fox emphasized, the homogeneity of surgical technique and cumulative familiarity constitute the fundamental prerequisites for the philosophy of uterus-preserving surgery to be successfully established in clinics [17].

Of the cases, 55.6% were taken to operation under elective and 44.4% under emergency conditions. The most frequent clinical picture leading to emergency surgery was vaginal bleeding, at 22.8%; 33.9% of the series delivered before the 37th gestational week, a rate consistent with the generally accepted late-preterm planned delivery strategy in the literature. Morlando et al. showed, in the IS-PAS database, that antepartum bleeding was the only independent predictor of emergency delivery (aOR 4.3) and that, when performed in expert centers, emergency delivery did not markedly increase maternal morbidity [21]. Regarding optimal delivery timing, the Pan-American study by Salmanian et al. found that maternal outcomes were largely comparable across different week windows; this finding emphasizes the importance of patient-specific decision-making rather than a “single correct time” approach [22]. In our series, the planning of the vast majority of elective cases between weeks 35–37 and the concentration of emergency cases in earlier weeks is in line with this international protocol trend.

The median operative time rose from 60 min during the 2013–2019 period to 90 min during the 2020–2025 period, reaching 120 min in cases undergoing IIAL, uterine lower segment resection, and hysterectomy. This paradoxical appearance should be interpreted as an inevitable “time cost” of the transition from radical surgeries to gradual and meticulous reconstructive techniques. The data support this interpretation directly: operative time increased in proportion to the number of uterus-preserving maneuvers applied, whereas in operations requiring no such maneuver the median operative time was identical in the early and late periods. The prolongation therefore reflects a deliberate change in surgical technique rather than any change in operating theater conditions or team composition. In the single-surgeon experience of Yalinkaya and Oglak, the mean operative time was reported as 54 min, a value consistent with the early-period time of our series [10]. In the low uterine segment sandwich technique developed by Caliskan et al., the combined application of systematic devascularization, balloon tamponade, and compression sutures was shown to prolong the operative time but to be a fundamental step in preventing organ loss [23]. In the expert review of Fox et al. focusing on the management of complex cesarean delivery, it was also stated that the stepwise application of lower segment dissection and vascular ligation increases the operative time, but that this prolongation represents a necessary surgical investment to limit major complications [24].

At least one blood product transfusion was administered to 56.5% of patients; the median erythrocyte suspension (ES) was three units, and the fresh frozen plasma (FFP) use rate was 44.4%. The most notable hematological finding of the series is that the use of recombinant activated factor VIIa (rFVIIa) was not required in any of the 324 cases. In the monocentric tertiary-center experience of Alhashim et al., it was emphasized that massive hemorrhage is the most prominent cause of morbidity in PAS cases and that proactive multiple-blood-product replacement should be integrated into the routine protocol [25]. Shamshirsaz et al. showed that an intraoperative blood loss of 1500 mL is a critical threshold value with a sensitivity of 80% and a specificity of 60% for the development of coagulopathy, and that below this threshold the risk of disseminated intravascular coagulation (DIC) remains minimal [26]. In the massive hemorrhage management protocols compiled by Park and Cho and in the case report by Laranjo et al., it is stated that expensive procoagulant agents such as rFVIIa should generally be reserved as “rescue” therapy in refractory coagulopathy [27,28]. In our series, the zero use of rFVIIa demonstrates that massive hemorrhage can be successfully controlled with systematic surgical devascularization, conventional blood product replacement initiated in a timely manner, and step-up hemostasis protocols.

The postoperative ICU admission rate was 8%, the median hospital stay was 2 days, and the reoperation rate was 1.2%. Erfani et al. reported a markedly higher ICU admission rate and blood loss in unexpected PAS cases compared with planned elective cases, and emphasized that the presence of an experienced team is more decisive than the depth of invasion [29]. While ICU admission rates in large PAS cohorts in the literature are generally reported between 20 and 40% [19], the 8% rate of our series reflects the effectiveness of preoperative prediction and intraoperative hemodynamic management. In the survey study by Shainker et al., it was reported that long hospital stays and traumatic intensive care processes adversely affect the psychosocial recovery of PAS patients and that post-traumatic stress disorder or depression was reported in 17% of patients [30]; this finding emphasizes that a short stay and a low complication profile are not merely a cost but a patient-centered, holistic clinical success.

At least one uterus-preserving surgical method was applied in 71% of cases, and hysterectomy was completely avoided in 88.9%. This rate is considerably above the in situ success reported in the multicenter IS-PAS database and the uterus-preservation rate in the van Beekhuizen series [16]; it confirms the critical role of high-volume single-surgeon experience in implementing this preserving paradigm. In the comprehensive expert opinion review of Fox et al., it was emphasized that conservative approaches should be evaluated on the basis of two fundamental goals: avoiding acute complications and preserving fertility [31]. Yalinkaya and Oglak reported that hysterectomy could be avoided in all 245 patients managed with modified conservative single-step surgery [10]. Lo et al. showed that an 80% uterus-preservation rate could be achieved even in a regional-level hospital with planned conservative approaches [32]. In our series, the steady clinical transformation observed especially after 2018 concretizes how this progressive organ-preservation philosophy is linearly strengthened by the surgeon’s cumulative experience.

In our series, the most frequently used uterus-preserving methods were, in order, the intrauterine hemostatic suture (IHS; 46.0%), internal iliac artery ligation (IIAL; 38.0%), Bakri balloon tamponade (34.3%), uterine lower segment resection (15.4%), and B-Lynch suture (4.0%), with the most frequently applied combination being the Bakri balloon together with the intrauterine hemostatic suture (8.6%), followed by the triple combination of Bakri balloon, IIAL and intrauterine hemostatic suture (6.2%) (Supplementary Table S1). This gradual “step-up” algorithm aligns with a stepwise management approach structured according to the severity of bleeding and its anatomical focus. In the low uterine segment sandwich technique described by Caliskan et al., the uterus was successfully preserved in all patients complicated by placenta percreta through the combined use of systematic devascularization, intrauterine balloon, and compression sutures [23]. Abousifein et al. emphasized the synergistic role of the simultaneous or sequential use of multiple hemostatic modalities in increasing surgical success [33]. In the resective–reconstructive technique described by Palacios-Jaraquemada, it was possible to avoid hysterectomy in approximately 80% of cases [34]. In their IS-PAS analysis, Schwickert et al. showed that focal resection offers an equivalent alternative to planned hysterectomy in terms of massive hemorrhage risk; this finding supports that reconstructive approaches are a reliable option in selected cases [35].

A total of 36 patients (11.1%) underwent peripartum hysterectomy; histopathological examination confirmed PAS in the forms of accreta, increta, or percreta in 75% of these cases. This high clinicopathological concordance rate reflects the accuracy of intraoperative surgical decision-making. In the hysterectomy group, age, operative time, blood product use, and length of hospital stay were significantly higher (*p* < 0.05); these clinical differences reveal that cases with the highest level of invasion depth and vascular complexity were, by their nature, concentrated in this group. In the 586-case series of Peng et al., it was shown that the tendency toward hysterectomy markedly increased as the depth of invasion increased [18]; Cetinkaya Demir et al., on the other hand, emphasized that hysterectomy rates could be lowered with cumulative experience in deep increta and percreta cases [19]. Erfani et al. reported that a timely decision for peripartum hysterectomy in unexpected massive hemorrhages is of absolute importance for maternal survival and that an experienced team is the decisive factor in this critical process [29]. In Fox’s editorial assessment, it was argued that comparative examination of single-step conservative surgery with planned cesarean hysterectomy constitutes the next scientific step in PAS management [31].

In our series, with an overall complication rate of 5.2% running quite low, the most frequent event was bladder perforation at 4.6%. Gastrointestinal injury was not observed in any case, and maternal mortality was recorded as zero. The rate of developing any complication in the hysterectomy group (22.2%) was approximately sevenfold higher than in the non-hysterectomy group (3.1%); bladder perforation was also markedly more frequent in this group, at 22.2%. In the literature, bladder injury rates in PAS cases are reported between 15 and 30%, and it is known that this complication shows a strong correlation with the depth of invasion and the degree of placental bed vascularity [19,28]. In the Cetinkaya Demir cohort, a 20% bladder injury rate was reported in the planned hysterectomy group; Erfani et al. emphasized the decisive role of preoperative preparation and surgical anatomical familiarity in limiting urological complications [19,29]. The 4.6% bladder perforation rate in our overall population is a concrete clinical indicator of the accumulation, over 13 years, of systematic dissection discipline repeated in the same hands and of stepwise uterine devascularization.

The perioperative hemoglobin change (ΔHb), accepted as one of the most objective surrogate parameters of actual maternal blood loss, was found to be a median of 1.7 g/dL in our series. This value closely coincides with the 1.5 g/dL decrease reported in the single-surgeon series of Yalinkaya and Oglak [10], and remains more limited than the mean 2.1 g/dL decrease in the Cetinkaya Demir series [19]. The relatively high values observed in the B-Lynch suture group (median ΔHb 2.6 g/dL) and the uterine lower segment resection group (2.1 g/dL) arise not from a failure of these techniques, but from the deliberate referral to these steps of the most complex and most severely hemorrhagic cases in which standard hemostatic methods were insufficient. As shown by Shamshirsaz et al., the risk of DIC remains minimal in bleeding kept below the 1500 mL threshold [26], and in our series the inclusion of the vast majority of these groups in the non-hysterectomy process confirms the effectiveness of the step-up escalation philosophy. In the analysis by Schwickert et al., the contribution of early and systematic devascularization to limiting total blood loss and perioperative Hb decrease was also emphasized [35].

Two features of the group comparison presented here require caution in interpretation. First, allocation to the hysterectomy and uterus-preserving groups was not predetermined but reflected the intraoperative course; women in whom hysterectomy was ultimately performed were, by definition, those whose bleeding proved refractory to conservative measures. The differences observed between the groups in operative time, transfusion requirement, hospital stay, and complication rate should therefore be read as descriptors of two distinct clinical trajectories rather than as evidence that one surgical strategy is superior to the other. Second, histopathological confirmation of placenta accreta spectrum is obtainable only from a resected uterus, so that the depth of placental invasion is known for the hysterectomy group alone and the two groups cannot be matched for this variable. Because invasion depth is among the strongest determinants of surgical difficulty, this represents an unavoidable selection bias inherent to any comparison of this kind within a uterus-preserving series, and it almost certainly contributes to the differences observed. The multivariable analysis was restricted to preoperatively available variables for the same reason, and its results should be interpreted with this residual confounding in mind.

### Strengths and Limitations

Among the strengths of the study are the long and uninterrupted 13-year follow-up, the elimination of inter-operator variation achieved by a single surgeon managing all cases, the achievement of zero maternal mortality, and the high histopathological confirmation rate in the hysterectomy group. As Braun et al. emphasized within the framework of the IS-PAS database, the heterogeneity arising from different teams in multicenter series constitutes the most fundamental methodological limitation [36]; in the present series, this factor has been completely eliminated. Nevertheless, several limitations must be acknowledged. The study is retrospective, single-center, and single-surgeon in design. Its retrospective nature constrained the data-recording process and left the histopathological classification incomplete in nine cases, and the FIGO clinical grading system could not be applied prospectively across the series, which limits the retrospective comparison of invasion depths and underlies the selection bias discussed above. The single-center, single-surgeon design eliminates inter-operator variability but simultaneously restricts external validity; as Yalinkaya and Oglak noted, outcomes achieved through the cumulative experience of one high-volume specialist may not be directly reproducible by teams of different experience levels [10]. No concurrent comparison group managed by an alternative strategy was available, so the temporal improvement observed cannot be formally attributed to the step-up approach alone: secular changes in transfusion practice, anesthetic management, antenatal imaging, and referral patterns occurred over the same period and may have contributed to it. Blood loss was estimated indirectly from the perioperative hemoglobin change rather than by direct volumetric measurement. The multivariable model was constrained by the small number of events (*n* = 36), which limited the number of covariates that could be entered and precluded external validation; its estimates should therefore be regarded as exploratory. Finally, subsequent fertility, menstrual function, and the outcomes of later pregnancies were not recorded, so the long-term benefit of uterine preservation—the principal justification for the approach—could not be assessed, and the psychosocial outcomes highlighted by Shainker et al. likewise could not be evaluated, although the short hospital stay and low complication profile indirectly support these holistic patient outcomes [30].

## 5. Conclusions

The 13-year single-surgeon experience presented in this study demonstrates that a standardized and gradual (“step-up”) uterus-preserving strategy can be applied in the management of placenta previa and placenta accreta spectrum cases with zero maternal mortality, limited perioperative blood loss, a low complication rate, and an exceptional uterus-preservation rate. Early antenatal diagnosis, systematic devascularization, controlled reconstruction of the placental bed, and the reinforcement of a hysterectomy-avoiding philosophy through cumulative experience constitute the cornerstones of modern PAS management. Our findings are consistent with evidence in the literature that surgical experience is a decisive determinant of outcome, and suggest that even in resource-limited centers these complex cases can be managed successfully through operative consistency and stepwise surgical algorithms. Given the retrospective, single-center design and the absence of a concurrent comparison group, these observations should be regarded as hypothesis-generating rather than confirmatory. Future prospective multicenter studies will contribute to determining the optimal surgical algorithm in PAS management by evaluating the reproducibility of this single-surgeon approach across a broader pool of practitioners.

## Figures and Tables

**Table 1:** Demographic, obstetric, and neonatal characteristics of the cohort (*n* = 324).

Characteristic	Median [IQR] or *n* (%)	Min–Max
**Baseline demographic and obstetric characteristics**		
Age (years)	32 [28–36]	19–50
Gravidity	3 [2–4]	1–10
Parity	2 [1–2]	0–6
Number of abortions	0 [0–1]	0–5
History of curettage	0 [0–0]	0–3
Number of vaginal deliveries	0 [0–0]	0–5
Number of previous cesarean sections	1 [0–2]	0–5
Gestational age at delivery (weeks)	37.4 [36.1–38.1]	21.5–40.2
**Mode of conception**		
Spontaneous	309 (95.4)	—
In vitro fertilization	14 (4.3)	—
Intrauterine insemination	1 (0.3)	—
**Indication for cesarean section**		
Elective	180 (55.6)	—
Emergency	144 (44.4)	—
**Neonatal outcomes (per fetus; *n* = 327)**		
Birth weight (g)	3010 [2645–3335]	435–4740
Apgar score at 1 min	7 [6–8]	0–10
Apgar score at 5 min	9 [8–9]	0–10
Neonatal mortality	1 (0.3)	—
**Maternal outcome (*n* = 324)**		
Maternal mortality	0 (0.0)	—

**Footnotes:** Continuous variables are presented as median [interquartile range] with the observed range, and categorical variables as number (percentage). Multiple pregnancies (*n* = 3) were handled on a per-fetus basis for the neonatal outcomes (*n* = 327 fetuses). IQR: interquartile range.

**Table 2:** Maternal intensive care, length of hospital stay, and reoperation (*n* = 324).

Characteristic	*n* (%) or Median [IQR]	Min–Max
**Maternal intensive care unit (ICU) requirement**		
No	298 (92.0)	—
Yes	26 (8.0)	—
**Reoperation**		
No	320 (98.8)	—
Yes	4 (1.2)	—
**Duration of care**		
ICU length of stay (days) ^a^	1 [1–2]	1–5
Postoperative hospital stay (days)	2 [2–3]	0–11

**Footnotes:** Categorical variables are presented as number (percentage) and continuous variables as median [interquartile range] with the observed range. ^a^ Calculated only for the 26 women admitted to the ICU. IQR: interquartile range; ICU: intensive care unit.

**Table 3:** Histopathological findings and clinical comparison according to performance of peripartum hysterectomy.

Characteristic	Hysterectomy (*n* = 36)	No Hysterectomy (*n* = 288)	*p*
**Histopathological findings in hysterectomy specimens**			
Placenta accreta	11 (30.6)	—	—
Placenta increta	11 (30.6)	—	—
Placenta percreta	5 (13.9)	—	—
Placenta accreta spectrum, total	27 (75.0)	—	—
Non-spectrum or indeterminate ^a^	9 (25.0)	—	—
**Clinical comparison between groups**			
Maternal age (years)	35 [31–38]	32 [28–36]	0.008
Gestational age at delivery (weeks)	37.6 [36.2–38.0]	37.3 [36.1–38.1]	0.932
Operative time (min)	120 [116–135]	60 [60–105]	<0.001
Total blood products (units)	6 [4–10]	1 [0–3]	<0.001
Postoperative hospital stay (days)	3 [3–5]	2 [2–3]	<0.001

**Footnotes:** Histopathological findings are presented as number (percentage) of the 36 women who underwent hysterectomy; continuous variables are presented as median [interquartile range] and were compared with the Mann–Whitney U test. ^a^ Cases in which the histopathological report did not confirm abnormal placental invasion or was inconclusive.

**Table 4:** Univariable and multivariable analysis of factors associated with failure of uterine preservation (peripartum hysterectomy) (*n* = 324).

Variable	Univariable Analysis	Multivariable Analysis		
	OR (95% CI)	*p*	OR (95% CI)	*p*
Maternal age (per year)	1.08 (1.01–1.15)	0.017	1.08 (1.00–1.16)	0.057
Previous cesarean sections (per section)	2.12 (1.58–2.84)	<0.001	2.41 (1.72–3.36)	<0.001
Gestational age (per week)	1.00 (0.85–1.18)	0.991	—	—
Emergency surgery (vs. elective)	1.00 (0.50–2.01)	1.000	—	—
Anterior placental location (vs posterior/lateral)	2.57 (0.94–7.02)	0.065	—	—
Total previa (vs. partial, marginal, or low-lying) ^a^	Not estimable	0.002	—	—
Surgical period 2020–2025 (vs. 2013–2019)	0.47 (0.22–1.01)	0.052	0.24 (0.10–0.59)	0.002

**Footnotes:** Binary logistic regression with peripartum hysterectomy as the dependent variable (36 events). Variables associated with the outcome at *p* < 0.10 on univariable analysis were considered for the multivariable model; the number of covariates was restricted to three in accordance with the events-per-variable principle, and the three showing the strongest univariable association were retained. Anterior placental location (univariable *p* = 0.065) was accordingly not included in the final model. Model performance: area under the receiver operating characteristic curve 0.814 (optimism-corrected 0.800 after 1000 bootstrap resamples); Hosmer–Lemeshow *p* = 0.676; bootstrap calibration slope 0.934. ^a^ All 36 hysterectomies occurred in women with total previa and none among the 71 women with partial, marginal, or low-lying previa; this complete separation precluded maximum-likelihood estimation, and the p value shown was obtained from the chi-square test. In a Firth penalized sensitivity analysis, total previa remained strongly associated with the outcome (OR 22.21, 95% CI 2.92–2852.36), with negligible change in the estimates for the other covariates. OR: odds ratio; CI: confidence interval.

## Data Availability

The raw data supporting the conclusions of this article will be made available by the authors on request.

## Supplementary Materials

The following supporting information can be downloaded at: https://www.mdpi.com/article/10.3390/medicina62081552/s1. Figure S1: Reconciliation of Group Sizes Across Figures (n = 324); Table S1: Uterus-Preserving Surgery: Frequency of Combinations Used (12 Most Frequent Combinations, *n* = 324).
