# Supplementary material for: Uterus-Preserving Surgery for Placenta Previa and Placenta Accreta Spectrum: Retrospective Analysis of the Evolution of a Standardized Step-Up Approach in a 13-Year Single-Surgeon Cohort of 324 Cases
Source: Medicina (Kaunas). 2026 Aug 12;62(8):1552. doi: 10.3390/medicina62081552 (PMC13515284; doi:10.3390/medicina62081552)
Supplement: Supplementary file 1 [file medicina-62-01552-s001.zip › medicina-4447926-supplementary.pdf]

Supplementary Materials

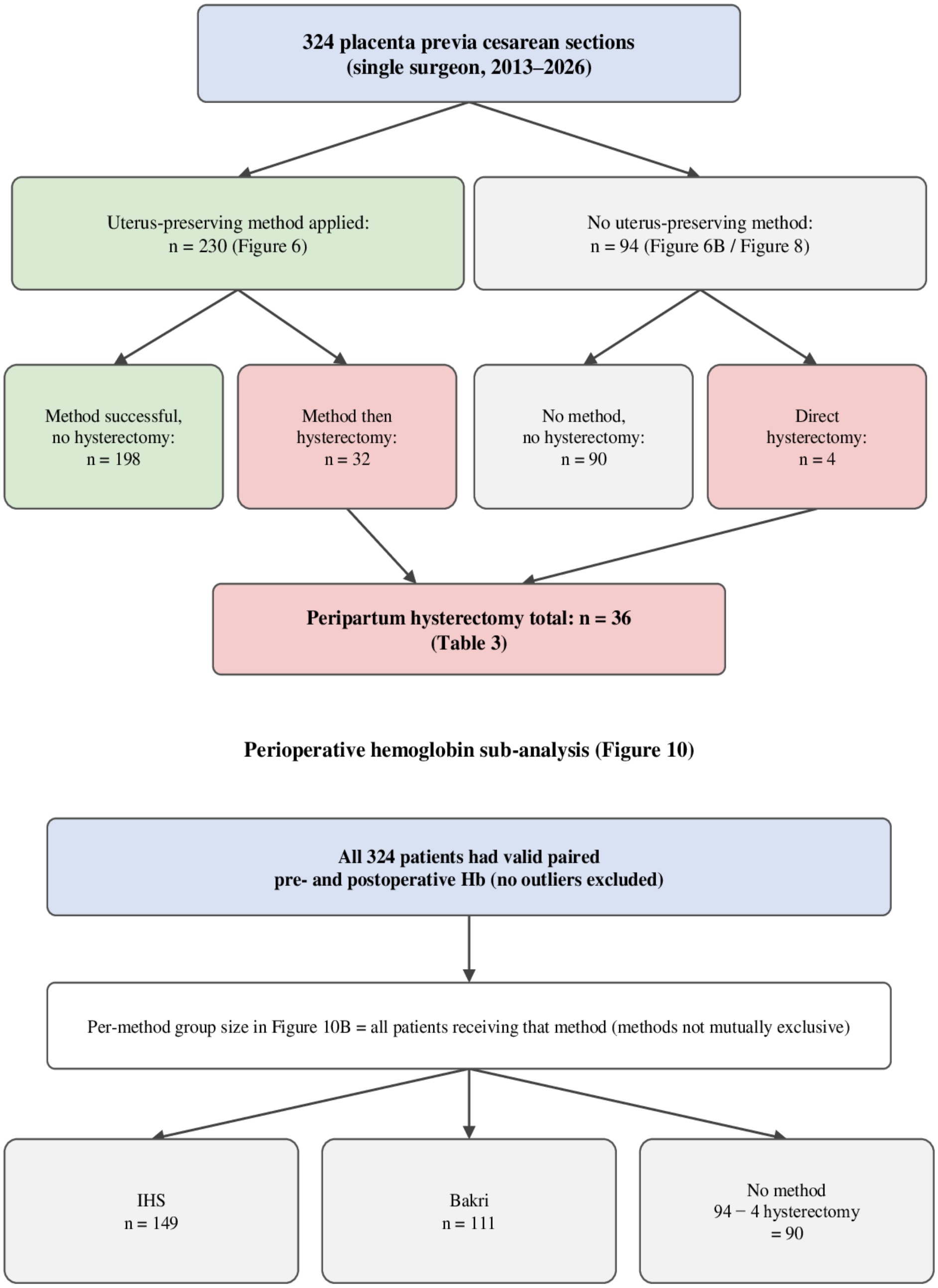

Figure S1. Reconciliation of Group Sizes Across Figures (n = 324)

Footnotes: *This diagram reconciles the patient numbers reported across Figures 6, 8, and 10. A uterus-preserving method was attempted in 230 patients and not used in 94. Of the 36 peripartum hysterectomies, 32 followed an attempted uterus-preserving method and 4 were performed directly. In the hemoglobin sub-analysis (Figure 10), all 324 patients had valid paired pre- and postoperative hemoglobin values. Because the methods are not mutually*

exclusive, each per-method group size in Figure 10B equals the total number of patients receiving that method (e.g., intrauterine hemostatic suture n = 149; Bakri balloon n = 111), and a patient receiving more than one method is counted in each respective group. The "No method" group (n = 90) corresponds to the 94 patients without any preserving method after excluding the four direct-hysterectomy cases (shown separately). IHS: intrauterine hemostatic suture.

Supplementary Table S1. Uterus-Preserving Surgery: Frequency of Combinations Used (12 Most Frequent Combinations, n=324)

Combination	Number (n)	Percentage (%)
Intrauterine hemostatic suture (alone)	44	13.6
IIAL (alone)	30	9.3
Bakri + intrauterine hemostatic suture	28	8.6
Bakri (alone)	22	6.8
Bakri + IIAL + intrauterine hemostatic suture	20	6.2
IIAL + intrauterine hemostatic suture + resection	18	5.6
Bakri + IIAL	13	4.0
Bakri + IIAL + intrauterine hemostatic suture + resection	13	4.0
IIAL + intrauterine hemostatic suture	9	2.8
IIAL + resection	8	2.5
Intrauterine hemostatic suture + resection	6	1.9
Bakri + IIAL + B-Lynch + intrauterine hemostatic suture	5	1.5

Footnotes: *IHS: intrauterine hemostatic suture; IIAL: hypogastric artery ligation. Only the 12 most frequent combinations are listed; rare combinations were omitted. Percentages were calculated relative to the total number of patients (n=324). This table should be evaluated together with Figure 6 and the related Results paragraph in the main text.*
